# Osteotomy Around the Knee: The Surgical Treatment of Osteoarthritis

**DOI:** 10.1111/os.13021

**Published:** 2021-06-10

**Authors:** Haining Peng, Aichun Ou, Xiaohong Huang, Chen Wang, Lei Wang, Tengbo Yu, Yingze Zhang, Yi Zhang

**Affiliations:** ^1^ Department of Sports Medicine Affiliated Hospital of Qingdao University Qingdao China; ^2^ Department of Operating Room Affiliated Hospital of Qingdao University Qingdao China; ^3^ Institute of Neuroregeneration and Neurorehabilitation, Qingdao University Qingdao China; ^4^ Institute of Sports Medicine and Rehabilitation, Qingdao University Qingdao China; ^5^ Department of Orthopedics The Third Hospital of Hebei Medical University Shijiazhuang China; ^6^ Department of Orthopedics Affiliated Hospital of Qingdao University Qingdao China; ^7^ Shandong Institute of Traumatic Orthopedics Affiliated Hospital of Qingdao University Qingdao China

**Keywords:** Distal femur osteotomy, mechanical axis, High tibial osteotomy, Knee osteoarthritis, Osteotomy around the knee joint, Proximal fibular osteotomy

## Abstract

Osteoarthritis causes joint pain and functional disorder, of which knee osteoarthritis is the most common. Nowadays, clinically effective treatments mainly include conservative treatment, arthroplasty, and osteotomy. However, conservative treatment only offers symptomatic relief and arthroplasty is limited to the patients with a moderate to severe degree of osteoarthritis. For relatively young patients who require greater knee preservation, a surgical treatment with low operation trauma and revision rate is needed. Osteotomy around the knee, based on the notion of “knee preservation,” has been chosen as an alternative surgical treatment. Cutting and realigning the bones corrects the mechanical line of lower limb force bearing. As such, osteotomy around the knee retains normal anatomical structure and obtains good functional recovery of the knee joint. The techniques of osteotomy around the knee includes anti‐varus deformity and anti‐valgus deformity osteotomy, aiming to reallocate the force bearing in the compartment of the knee joint. By choosing the surgical section of the lower limbs, the osteotomy around the knee can achieve the correction of mechanical axis, such as the high tibial osteotomy (HTO), proximal fibular osteotomy (PFO), and distal femur osteotomy (DFO). Numerous modified techniques have been developed to meet the demands of patients based on traditional methods. These modified osteotomy have their own advantages and indications. This paper aims to guide clinical treatment by reviewing different types of osteotomies, and their effects, that have been studied and applied widely in clinical practices.

## Graphical abstract

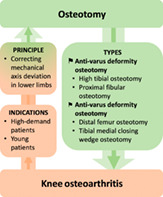

Osteotomy around the knee (OAK), one of the clinical treatments for knee osteoarthritis (OA), is based on the notion of “knee preservation” and primarily applies to relatively young and high‐demand patients. The principle of OAK is to correct the mechanical axis in the lower limbs. According to the types of deformity, the techniques can be divided into anti‐varus deformity and anti‐valgus deformity osteotomy. By choosing the surgical section of the lower limbs, anti‐varus deformity and anti‐valgus deformity osteotomy can be further divided into high tibial osteotomy (HTO), proximal fibular osteotomy (PFO), and distal femur osteotomy (DFO). Osteotomy is easy to conduct, low‐risk, almost all cases have rapid recovery after surgery, and it has been chosen as an alternative surgical treatment.

## Introduction

Knee osteoarthritis (KOA) is a common chronic disease causing joint pain and functional disorders in 30% of >60‐year‐old people^1^, ^2^. In addition to age and obesity, the abnormal mechanical factor plays a critical role in the pathogenesis of osteoarthritis^3^.The mechanical axis deviation in the lower limbs causes the varus or valgus deformity, which affects the load‐bearing force of the medial and lateral knee joint compartments, and increases the pressure held by the cartilage and subchondral bones. This in turn accelerates the mechanical axis deviation in lower limbs, which eventually worsens the progress of osteoarthritis. The principle of osteotomy around the knee (OAK) is to rebalance the force between the medial and lateral compartments, reduce the pressure borne of the cartilage and subchondral bones by correcting the mechanical axis with bone cutting^4^. OAK can relieve joint pain and improve joint function. Due to the fact that osteotomy retains the anatomical structure of the joint, it has the advantages of proprioception preservation and rapid recovery of joint functional efficacy, which greatly delays the progression of osteoarthritis. OAK thus better suits relatively young and high‐demand patients.

**Fig 1:**
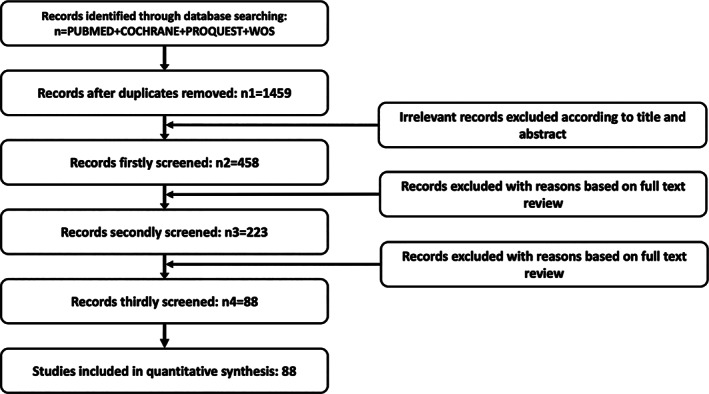
Study selection process. Preferred reporting articles for this review flow diagram.

There are many techniques for correcting the mechanical axis of OAK. We conducted a structured literature search in the PubMed, Cochrane, ProQuest, and Web of Science, to identify studies published between January 1965 and 2020 using the search string “knee osteoarthritis” or “varus deformity” or “valgus deformity” or “gonarthrosis” or “mechanical axis” AND “high tibial osteotomy” or “proximal fibular osteotomy” or “distal femur osteotomy” or “osteotomy around the knee joint.” We manually reviewed reference lists in all retrieved articles for related publications (Fig. 1).

Inclusion criteria were: studies investigating either osteotomy around the knee joint and/or osteotomy; studies reporting original human data; and studies that introduced relevant techniques and modified techniques. Exclusion criteria were: animal studies; cellular studies; studies with less than three subjects per group; case reports; letters to the editor and editorials; and publications not reporting original data. The aim of this paper is to provide an overall evaluation for clinical treatments through reviewing the different types of OAK that have been reported in clinical practices for treating knee osteoarthritis.

## Anti‐varus Deformity Osteotomy

Biomechanically, 60%–80% of load bearing is transmitted to the medial compartment of the knee joint in normal gait^5^. And the involvement degree of the medial compartment is 10 times that of the lateral compartment^6^. Therefore, medial compartment osteoarthritis with varus alignment and medial space stenosis is more common compared to lateral compartment osteoarthritis, accounting for about 74% of patients with KOA^7^. To reallocate the force bearing from the medial to the lateral compartment,the anti‐varus osteotomy, including high tibial osteotomy (HTO) and proximal fibular osteotomy (PFO), corrects the mechanical lines of lower extremities using surgical methods, which alleviates the abrasion of medial cartilage and relieves pain^8^.

### High Tibial Osteotomy (HTO)

High tibial osteotomy (HTO) is an accepted surgical treatment in medial compartment arthritis^8^, ^9^, ^10^. An effective ball‐and‐socket osteotomy below the tibial tubercle has been reported by Jackson and Waugh^11^ as significantly improving the postoperative survival rate (cumulative survival with conversion to arthroplasty)^12^, ^13^. The classical approach of HTO has some complications, including neurovascular injury, under correction, and facture^14^, ^15^, ^16^, yet improvement in the surgical techniques has earnt HTO widespread attention in recent years.The strategy for correcting the medial compartment arthritis with varus deformity is based on cartilage non‐progressive injury area^17^, i.e. 30%–40% of the lateral tibial plateau. It is recommended that 62.5% away from inner side of the tibial plateau (Fujisawa point) should be the aiming point for correcting the mechanical axis^18^.

The operation of HTO needs to consider the age of patients and their functional requirements, the position and severity of knee joint deformity, and the progression of the disease:

1. Age: HTO, reserving the anatomical structure of the knee joint, is more important for people who need joint preservation, especially relatively young patients. The surgery is usually recommended for patients less than 60 years of age^19^.
2. Varus deformity: It has been indicated in the previous studies that the postoperative consequence is better when the tibia bone varus angle (TBVA) is more than 5° or the medial proximal tibial angle (MPTA) is less than 85°^19^.
3. The disease progression: The degree of cartilage abrasion does not affect the correction outcome of osteotomy^20^. However, it is difficult for patients with severe subchondral bone abrasion to achieve satisfactoriness after HTO.

#### Medial Open‐Wedge High Tibial Osteotomy

The methodology of medial open‐wedge high tibial osteotomy (MOWHTO)^21^ is to perform oblique cutting at the medial side of the tibia and implant bones in the gap after the wedge is extended. It is necessary to keep the lateral tibial cortex intact. The 10‐year survival rate of MOWHTO is as high as 91.6% and the life‐long survival rate is over 65%^22^. Early applications of the surgery entail a single‐plane osteotomy, which affects the patella position and the tibia posterior tilt in the sagittal plane. Therefore, efforts have been made for long‐term and stable treatment results, like the different approaches of osteotomy, the stabilizing devices, and the newly implanted materials^24^, ^25^, ^26^. The modified techniques, including dual‐plane osteotomy, fixation devices, the external fixator, and the absorbable mesh gasket, are developed in response to improve the effectiveness of the procedure.

##### Dual‐Plane High Tibial Osteotomy

**Fig 2:**
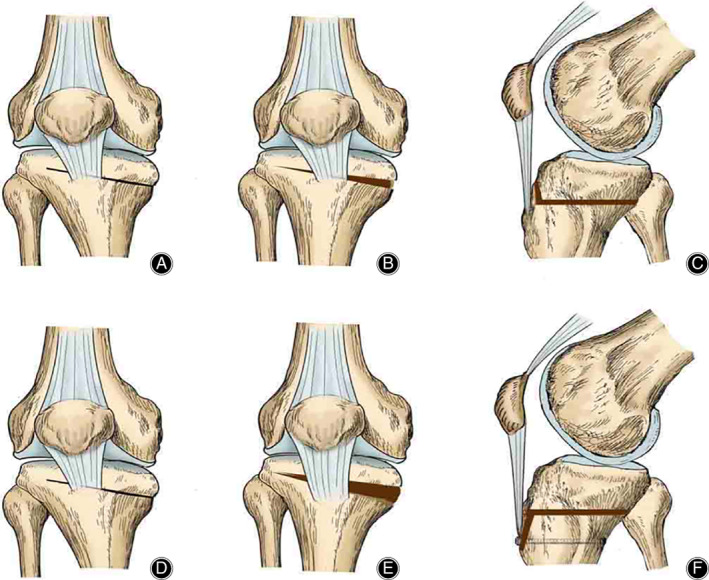
Medial open‐wedge high tibial osteotomy (MOWHTO) with proximal tibial tuberosity (PTO) and MOWHTO with distal tuberosity osteotomy (DTO). The a‐c indicate the methodology of MOWHTO with PTO. (A) before opening of the wedge, AP‐view; (B) after opening of the wedge, AP‐view; (C) after opening, lateral view. The d‐f indicate the methodology of MOWHTO with DTO. (D) before opening of the wedge, AP‐view; (E) after opening of the wedge, AP‐view; (F) after opening, lateral view. The figure was adapted from Gaasbeek *et al*.^24^

Early practices of dual‐plane high tibial osteotomy, also known as the superior incision, were performed at the proximal tibial tuberosity (PTO) (Fig. 2), employing a horizontal plane incision (cutting off the inner and posterior cortex of tibia) and an oblique incision behind the tuberosity in the coronal plane (cutting off the anterior cortex of tibia), whereby the angle between the two cut lines is about 110°. The upper incision method prevents the tibia from rotating. However, the patellar descending, causing pain in anterior knee and limiting joint function, remains a problem.

The subsection method, by modifying the operation and practicing distal tuberosity osteotomy (DTO) (Fig. 2), keeps the tuberosity attached to the proximal tibia^24^. After internal fixation of the osteotomy, a bicortical screw is used to fix the distal tuberosity to the tibia. The subsection procedure effectively prevents post‐osteotomy patellar from descending, which overcomes the shortage of superior incision, especially in patients who need a major anti‐varus deformity correction for medial compartment osteoarthritis.

##### Fixation Device

HTO requires higher internal fixation to maintain joint stability. However, classical plates hardly meet the need, which results in losing the corrected angle after the operation^24^, ^27^. To overcome the difficulty, different fixation techniques were described. The Puddu plate and TomoFix are the two most commonly used devices^27^. The original Puddu plate^28^ was described as a fixation device including the plate, screw, and a metallic block configuration, for distracting medial corticalis and supporting extra pressure^27^. The first generation Puddu plate can withstand the axial loading of the proximal tibia^29^. The modified one through LHS holes makes orientating the screws possible^30^.

TomoFix, a new locking compression plate (LPC), is a T‐shape plate including horizontally locking screw holes and longitudinally combining holes^26^. TomoFix plates have better stability and elasticity to maintain the correction without using bone to fill the gap. The excellent stability of the LPC can upgrade the postoperative load bearing, meanwhile the elasticity augments the contact between bones and facilitates bone healing^31^.

The finite element analysis (FEA) is widely accepted in data research^33^, ^34^. Some previous FEA compared different plates, the researchers concluded that the TomoFix plate produces superior compression and torsion stability than Puddu plate^28^, ^35^. Current literature mostly indicates that TomoFix plate is the optimal choice for internal fixation.

##### Ilizarovtype Circular External Fixator

Ilizarovtype circular external fixation was developed to render better rotational stability and rapid fullweight bearing for the joint^35^. Cengiz *et al*.^36^ indicate that the external fixation maintains precise correction and bone stock after surgery. However, it has a high risk of pin‐track infection^36^, ^38^ and is not widely used in clinical practice. Comparing the effects between internal and external fixation on postoperative stability, the internal fixation is believed to have a higher whole stability and a lower infection risk, and it is recommended in clinical application.

##### High Tibial Osteotomy Embedded in Absorbable Mesh Gasket

Several problems in HTO have been exposed in the early practices, such as complete fracture of tibia and secondary operation to remove internal fixation. Yingze Zhang *et al*.^25^ first used the technique of propping up the medial platform, which uses an absorbable gasket material, combined with a lateral fibular osteotomy to treat medial compartment arthritis. It has been reported that the knee joint function of the postoperative patients can be favorably improved within 6–12 months, with pain relief and fewer complications. The absorbable gasket is mainly composed of hydroxyapatite and collagen. The pores of the material are conducive for bone healing and growth, and the barbs can prevent the gasket from slipping out the gap. Moreover, the absorbability of the gasket prevents the trauma by secondary removal operations.The modified technique maintains the lateral tibial cortex which belongs to the incomplete fracture. Therefore, the procedure does not obstruct early postoperative weight bearing. The absorbable pads are reported to achieve best satisfactory improvement for knee osteoarthritis patients who have varus deformity between 10° and 15°^38^.

#### Lateral Closing Wedge High Tibial Osteotomy

Lateral closing wedge high tibial osteotomy (LCWHTO) was first invited by Coventry^39^, which has been the standard method for many years. By removing a wedge‐shaped bone block laterally, retaining the inner hinge, and closing the gap, the procedure effectively relieves the symptom of pain and improves joint mobility functions combined with proximal fibular osteotomy. Its 20‐year survival rate achieves 80% success. It is noteworthy that, especially for young patients, the joint stability is immediately improved and the healing time is shortened^41^, ^42^, ^43^. However, several complications have been reported in procedure, including nerve injuries, bone nonunion, and infection^23^. Duivenvoorden *et al*.^43^ report that around 4% of the postoperative patients have peroneal nerve palsy. Therefore, modification of surgical techniques is still needed to minimize the adverse effects.

##### Improved Lateral Closing High Tibial Osteotomy

The traditional LCWHTO affects the biomechanics of the patellofemoral joint, decreasing the tibia posterior tilt by approximately 5°, which results in increased force bearing of the cruciate ligament^44^. Huang *et al*. improved the surgery by making the spot of osteotomy in the distal tibial tubercle instead of the proximal tibial tuberosity, and using TomoFix plate for internal fixation^45^. It has been indicated that the modified surgery avoids the adverse impact on the patellofemoral joint movement and the reduction of the tibial slope, which effectively relieves postoperative pain and other symptoms. At present, this method is commonly applied to young patients and patients with excessive knee varus deformity.

#### Dome‐Shaped High Tibial Osteotomy

**Fig 3:**
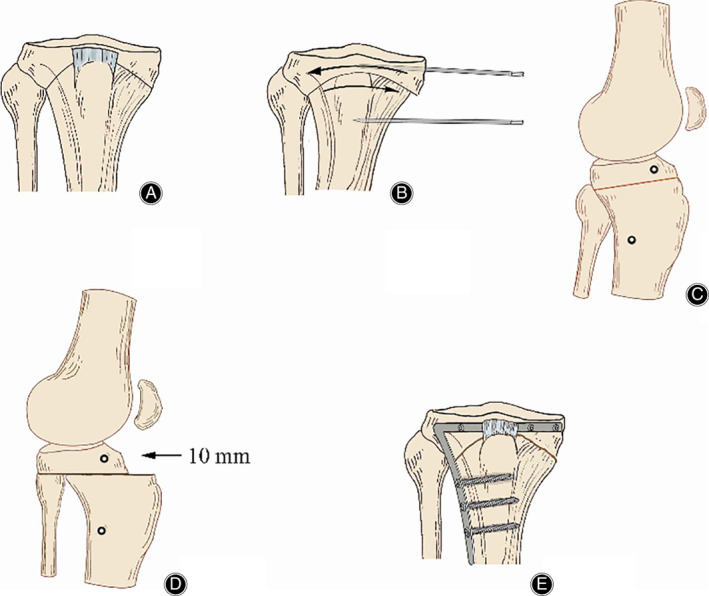
The method dome‐shaped hightibial osteotomy. (A) Series of 2.5‐mm drill holes marked on a curved line above the tibial tuberosity. (B) Two Steinmann pins inserted on either side of the osteotomy to define angular correction. (C/D) Sagittal view: the distal tibia brought forward approximately 10 mm. (E) When desired angle is achieved, the TomoFix plate fix fragments are applied under compression. The figure was adapted from Diogo *et al*.^47^

Compared with wedge‐shaped osteotomy, dome‐shaped osteotomy preserves the natural shape of the proximal tibia, maintains the length of lower limbs, and shortens the bone‐healing period, without affecting the joint replacement surgery afterwards. The method was invited and laterally popularized^46^. Dome‐shaped high tibial osteotomy (DSHTO) (Fig. 3) is used to correct the mechanical axis of lower limbs by removing a marginal amount of bones to generate rotation effect. Different from the linear displacement correction method in wedge osteotomy, DSHTO minimizes distance alteration between tibial tubercle and joint line, whereby, it better maintains the position of patella. Even to the young patients who have failed in LCWHTO correction,the application of DSHTO surgery can achieve improvement^47^. However, due to the large intraoperative exposure and complicated surgical techniques, DSHTO is rarely used in clinical practice.

#### L‐Shaped High Tibial Osteotomy

**Fig 4:**
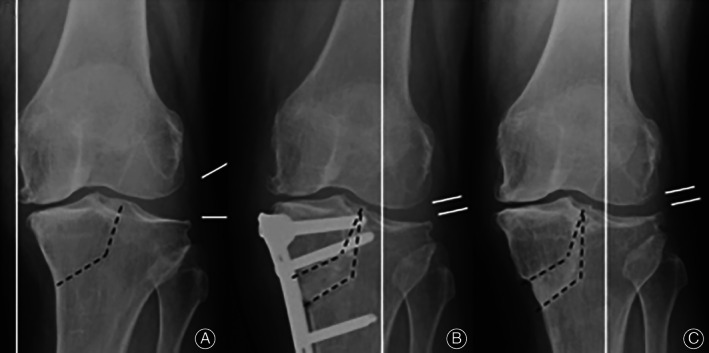
The methodology of the L‐shaped high tibial osteotomy. (A) an L‐shaped osteotomy from the medial side of the proximal tibia to the intercondylar eminence. (B,C) the weight‐bearing line is shifted out and the lateral subluxation joint is reset after correction. The figure was adapted from Chiba *et al*.^48^

L‐shaped high tibial osteotomy (LHTO), known as tibial condyle valgus osteotomy (TCVO), is an open intra‐articular osteotomy, which was developed by Chiba *et al* ^48^.The “L‐shaped” part of the term refers to the section from the proximal tibia to the intercondylar spine (Fig. 4). The procedure aims to contact tibia with the lateral femoral condyle after the osteotomy. The advantage of LHTO is multi‐operations, i.e. it allows more than one multi‐dimensional and multi‐planar corrections all at one time. Its effectiveness has been confirmed in many studies, including correct joint instability and lower limb alignment^48^. TCVO is applied to patients with intra‐articular deformity in the middle and late stages. However, experts claim that classical extra‐articular osteotomy technique cannot correct intra‐articular deformities^49^. TCVO also has a limited angle of varus correction. It corrects the tibia from valgus deformity into lateral joint reduction, rarely changing to the mechanical lines of the entire lower limb or the surrounding soft tissues, whereby the imbalanced tension between internal and external soft tissues remain unchanged.

#### Navigated Knee Osteotomy

HTO is a technically demanding, highly accurate procedure. The clinic results of HTO also depends on the degree of the accurate correction^15^. The failure may occur if the postoperative malalignment is unreliable. As in the previous studies, some computer‐assisted surgery (CAS) technologies have been used in preoperative planning^51^, ^52^. These can help to simulate surgery and predict possible outcomes^50^. However, there are not enough satisfying ways to achieve the mechanical axis intraoperatively.

The insufficient intraoperative visualization results in postoperative malalignment and surgical failure^52^. Meanwhile, exact intraoperative mechanical axis is a tough clinical problem. Laterally, the intraoperative system has been developed to help the intraoperative procedure^54^, ^55^. The technique of momentary evaluation includes the cable method which uses diathermy cable to span, the grids with leading impregnated reference lines and the radiologic measurement^56^, ^57^, ^58^.

Nowadays, the usefulness of the navigated system in HTO is gradually attracting attention. Ellis *et al*. first developed software that increased the accuracy of removing wedge intraoperatively with the need of preoperative CT scan^53^. With the awareness of radiation exposure and infection^58^, the CT‐free OrthoPilot® system was developed. The navigation has reported successful outcomes^59^. It can effectively prevent the loss of correction and increase the accuracy of intraoperative alignment^60^.

The preoperative planning and the intraoperative system are related long‐term outcomes. The way of taking a radiograph in the preoperative planning stage can result in the difference of accuracy^61^.

### Proximal Fibular Osteotomy (PFO)

Proximal fibular osteotomy (PFO) is based on the “non‐uniform settlement” theory proposed by Yingze Zhang *et al*.^62^.It indicates that osteoporosis triggers the non‐uniform settlement and degeneration, by decreasing the number of bone trabeculae and the ability of bone to disperse pressure leading to microfracture^62^. It is to cut small section of the proximal fibula, i.e. below the fibula head, which breaks the fibula and weakens its support for the lateral of the tibial plateau. As such, the muscle attached to proximal fibula, in the situation of the weight‐bearing, can pull the fibular head along the distal direction, and the tension is transmitted to the lateral femoral condyle. Eventually, the gap on the lateral side of the knee joint is reduced to offset the knee varus deformity caused by weight bearing. With the support of fibula on the lateral side, the load bearing axis shifts to the medial compartment, accelerating the settlement rate of the medial platform^63^. It is the reason that PFO was brought up to treat medial knee osteoarthritis^64^. Liu *et al*.^65^ revealed the relationship between preoperational factors and postoperative outcome: the outcome of PFO is related to the value of the settlement which reflects the supporting effect of the lateral fibula. Previous studies indicate that PFO can increase femorotibial angle by 1° to 5°, meanwhile the pressure on medial knee joint can be reduced by 10% to 30%^66^. According to the Kellygren–Lawrence's classification of knee arthritis, patients with knee arthritis grades II and III are recommended PFO surgery^68^, ^69^. Therefore, PFO is mainly considered for patients with early knee varus deformity and medial space stenosis.

PFO is a simple, trauma‐minimized, and effective procedure that enables patients to perform rehabilitation exercises and bear weight at earlier postoperative stage^69^. Thus, PFO is widely used in recent decades. However, PFO surgery may destroy the peroneal nerve, accompanied with the clinical manifestations, including the weakness of the dorsal extension of foot and numbness and decreased sensation on the back of foot and ipsilateral lower leg. However, the side effect can be gradually relieved by symptomatic treatment^70^.

## Anti‐valgus Deformity Osteotomy

Lateral compartment gonarthrosis which leads to valgus deformity accounts for about 16% of knee arthritis cases. The causing reason includes lateral meniscus injury, obesity, and so on. Valgus deformity reduces the knee adduction ability, causing the joint weight bearing to move towards the lateral compartment^71^. Anti‐valgus deformity osteotomy maneuvers the load bearing to the medial side to correct the force line of lower limbs, thereby, it rebalances the load bearing among different compartments. Anti‐valgus deformity is to settle the load bearing point in the 48%–50% area of the tibial plateau width from medially to laterally. Moreover, the corrected angle of the osteotomy is formed by the straight lines from the femoral head and the center of the talus to 50% of the tibial plateau, following the correction plan designed by Dugdale *et al*.^72^ It is suitable for young patients with earlier lateral knee osteoarthritis and lateral femoral condyle cartilage injury.

### Distalfemur Osteotomy (DFO)

Distalfemur osteotomy (DFO) was recommended as an alternative treatment for lateral osteoarthritis^73^, the correction osteotomy is usually applied locally where the deformity occurs. However, studies indicate that when the valgus deformity exceeds 12° or the joint surface deviates from the horizon to more than 10°^74^, the deformity should be corrected on the femur site, even if the deformity occurs on the tibia. Otherwise, it is more likely for the tibia to subluxate laterally, which causes knee joint instability^75^.

#### Distal Femoral Lateral Open‐Wedge Osteotomy

The lateral open wedge osteotomy, first proposed by Puddu *et al*.^76^, adjusts the lower limb force line based on the gap size and uses internal fixation to stabilize the osteotomy site. It maintains the corrected angle, and avoids shortening the lower limbs^77^. The procedure is suitable for young patients with knee valgus and loose ligaments. Moreover, it has been indicated in many studies that the lateral open surgery has a stable outcome with a 10‐year survival rate at around 74%^79^, ^80^, ^81^. However, the main problems are the fixed plate which irritates the iliotibial band and the long bone healing time. Jacobi and his colleagues^81^ studied the postoperative complications of the lateral open wedge osteotomy. They found that it took 9 months for bone to knit together properly in one case, and 86% of the patients have symptoms of iliotibial band irritation.

#### Distal Femoral Medial Closing Wedge Osteotomy

The closed wedge‐shaped osteotomy of the medial femur was first proposed and popularized to the clinical application by Coventry^74^. The line of force were corrected by cutting the wedge‐shaped bone block off the medial femur. The bone healing time is shortened due to the compression fixation plates, and the irritation of soft tissues is reduced by the medial approach.

Backstein *et al*.^82^ exemplify the complete recovery of patients' knee function after distal femoral lateral open‐wedge osteotomy, and the postoperative 10‐year survival rate can reach 87%^84^, ^85^. However, the correction angle depends on the size of wedge‐shaped bone block^85^. The corrective accuracy is limited owing to the precise bone wedge and the medial approach. The medial wedge‐shaped osteotomy may cause undesirable consequences such as the leg length discrepancy. This surgery, nevertheless, is commonly used in clinical application, as it avoids the complications in lateral open surgery.

### Tibial Medial Closing Wedge Osteotomy

In addition to distal femoral lateral open‐wedge osteotomy, Coventry^75^ has proposed the proximal tibial osteotomy as another treatment to correct valgus deformity. By realigning the proximal tibia and releasing the pressure of lateral compartment, the surgery corrects deformity, reduces cartilage damage, and relieves pain. Osteotomy can be applied on the tibia when the deformity occurs on the tibia or the joint coronal surface tilt is less than 10°. Collins *et al*.^86^ mention that, for small‐angle deformity, proximal tibial osteotomy warrants better outcomes. Owing to its large area of bone contact, postoperative gap of the bone heals within 4 weeks, and weight bearing function can be restored at early postoperative phase. It is notable that there is less complication compared to others surgical osteotomies.

## Conclusion

Osteotomy around knee joint advocates the concept of step‐up treatment. It is an effective treatment for knee osteoarthritis and has unique advantages compared to knee arthroplasty. Knee arthroplasty is at a high risk of postoperative infection. What's worse, younger patients who have had the replacement may need repeated revisional surgery afterwards^87^. Osteotomy is easily operated, low‐risk, and almost all cases obtain rapid recovery after surgery, which is comparable to arhroplasty. We have reviewed the advantages and disadvantages of different types of osteotomy around the knee (Table 1), and aim to provide guidance and support in clinic. Though skepticism indicates that the long‐term effect of osteotomy around knee joint is still uncertain, with the development of the upgraded equipment and advanced techniques, this approach can be improved and become more accurate, effective, and convenient.

**TABLE 1:** The summary of different types of osteotomy around the knee

Style	Advantages	Disadvantages	References
HTO	1. Standardization 2. Various types	1. Neurovascular complications 2. Facture 3. Delayed union and nonunion 4. Infection 5. Thromboembolic disease 6. Compartment syndrome 7. Under correction and recurrence of deformity	Aglietti*et al*. ^14^ Sprenger *et al*. ^15^ Wu *et al*. ^16^ Kim *et al*. ^22^ Coventry *et al*. ^40^, ^76^
PFO	1. Simple 2. Trauma‐minimized	1. Neurovascular complications 2. Compartment syndrome 3. Delayed union and nonunion 4. Conversion to total knee arthroplasty	Dong *et al*. ^63^ Zhang *et al*. ^64^ Yang *et al*. ^70^ Wang *et al*. ^69^
DFO	1. Accurate correction 2. Not affect the range of motion	1. Iliotibial band irritatio 2. Leg length discrepancy 3. Delayed union and nonunion	Puddu *et al*. ^77^ Dewilde *et al*. ^78^ Saithna *et al*. ^80^ Jacobi *et al*. ^81^ Marti *et al*. ^85^

DFO, distal femur osteotomy; HTO, high tibial osteotomy; PFO, proximal fibular osteotomy.
